# Short Communication: Awareness of HIV Self-Care Interventions Across Global Regions: Results from a Values and Preferences Survey

**DOI:** 10.1089/aid.2021.0200

**Published:** 2022-08-05

**Authors:** Kalonde Malama, Carmen H. Logie, Manjulaa Narasimhan, Léopold Ouedraogo, Chilanga Asmani, Hafya Elamin, L. Leigh-Ann van de Merwe, Jonathan Hopkins, Elizabeth Anne Bukusi

**Affiliations:** ^1^ Factor-Inwentash Faculty of Social Work, University of Toronto, Toronto, Canada.; ^2^ Department of Sexual and Reproductive Health and Research, Includes the UNDP/UNFPA/UNICEF/WHO/World Bank Special Programme of Research, Development and Research Training in Human Reproduction—HRP, Geneva, Switzerland, World Health Organization, Geneva, Switzerland.; ^3^ World Health Organization Regional Office for Africa, Brazzaville, Republic of Congo.; ^4^ Reproductive, Maternal Health and Ageing Team, Intercountry Support Team for West Africa, World Health Organization, Ouagadougou, Burkina Faso.; ^5^ Reproductive, Maternal Health and Ageing Team, Intercountry Support Team for East and Southern Africa, World Health Organization, Harare, Zimbabwe.; ^6^ Social Health Empowerment, Feminist Collective of Transgender Women of Africa, East London, South Africa.; ^7^ U-Turn, Cape Town, South Africa.; ^8^ Center for Microbiology Research, Kenya Medical Research Institute (KEMRI), Nairobi, Kenya.; ^9^ Department of Global Health and Obstetrics and Gynecology, University of Washington, Seattle, Washington, USA.

**Keywords:** self-care, HIV, Africa, universal health coverage

## Abstract

The high burden of HIV in sub-Saharan Africa places significant demands on health care services. Interventions such as HIV self-testing, and pre- and post-exposure prophylaxis (PrEP and PEP) could empower individuals to determine their HIV status and prevent HIV acquisition. In 2018, the World Health Organization disseminated an online, anonymous, global values and preferences survey to adults 18 years of age and older. The survey aimed to inform guidance on awareness, use, and preferences around self-care interventions for sexual and reproductive health. We conducted a cross-sectional analysis using Pearson's chi-squared test to compare awareness of HIV self-testing, PrEP and PEP across five global regions. Our analysis included 814 participants from 110 countries. We noted that respondents from Africa reported higher awareness of HIV interventions than participants from Europe, Latin America and the Caribbean, North America, and Asia. Our finding highlights an opportunity to expand self-care interventions for HIV prevention and management in Africa.

Self-care interventions include evidence-based, quality medicines, diagnostics, devices, and digital technologies that can be provided fully or partially outside of formal health care services and have the potential to increase universal health coverage.^1^ The high burden of HIV in sub-Saharan Africa places significant demands on health care services. Interventions such as HIV self-testing, and pre- and post-exposure prophylaxis (PrEP and PEP) could empower individuals to determine their HIV status and prevent HIV acquisition.^2–4^ Self-care interventions for HIV prevention have the potential to decentralize health care and circumvent overcrowding at health facilities when distributed, for instance, by community-based health workers or over-the-counter at pharmacies.^5^

With around two-thirds of people living with HIV being in sub-Saharan Africa, it is worth comparing whether the diffusion of HIV interventions matches the disproportionate burden of HIV in the Africa region compared to less endemic global regions. HIV prevalence in Eastern and Southern Africa (6.7%) and West and Central Africa (1.4%) considerably outweighs the regional prevalence in the Caribbean (1.1%), Latin America (0.4%), Europe, and North America (0.2%) and Asia (0.2%).^6^ Despite sub-Saharan Africa shouldering the highest geographical burden of HIV, it is unclear whether this disease burden is aligned with knowledge of HIV self-care interventions among laypersons and health care professionals. We thus examined how Africa compares to less HIV-endemic regions vis-à-vis awareness of HIV self-testing and PrEP and PEP.

Between July and November 2018, an online cross-sectional global values and preferences survey was disseminated to laypersons/potential end-users of self-care interventions and health care professionals. The survey was designed to inform the development of the World Health Organization's (WHO) global normative guidance, focused on awareness, use, and preferences around self-care interventions for sexual and reproductive health and rights, including HIV.^7^ The survey was developed by the WHO's Department of Sexual and Reproductive Health, who distributed it through their website, and 35 sexual and reproductive health listservs, where email addresses of sexual and reproductive organizations were stored. A sample size of 1000 respondents was estimated to be necessary, based on calculations to achieve regional representation across the various countries that participants came from.

Inclusion criteria were being 18 years of age and older, being able to read and write in any of the target languages of the survey, and being willing and able to provide informed consent. Upon receiving the survey, individuals chose whether or not to participate in it. Those who decided to participate provided informed consent before completing the survey in their choice of English, French, or Spanish. Surveys were completed anonymously and all questions were optional to minimize response bias. No monetary compensation was offered to participants in the online survey. Research Ethics Board approval was obtained from the University of Toronto, Canada (Protocol 36022).

Our primary response variable for the analysis was awareness of HIV self-testing and PrEP and PEP, determined by asking participants, “have you heard of HIV self-testing/PrEP/PEP (yes or no)?” Due to the binary nature of response variables, we used Pearson's chi-squared test to compare awareness of HIV self-testing and PrEP and PEP across five global regions. We specifically compared findings from the Africa region to those of four other regions (North America, Latin America and the Caribbean, Europe, and Asia). We used a statistical significance level of *p* < .05 to determine differences in awareness of HIV interventions between regions. Summary statistics performed included frequencies and percentages for categorical variables and median and interquartile range for continuous variables (*i.e.,* age), which were not normally distributed. All analyses were conducted in Stata 16 (StataCorp, College Station, Texas).

Our analysis included 814 participants from 110 countries. The median age of participants was 31 years (interquartile range: 23–43). The majority of participants who disclosed their gender were women (*n* = 557, 69%), with the rest identifying as men (*n* = 248, 30%) and transgender (*n* = 7, 1%). Europe (*n* = 224, 27%) and Africa (*n* = 215, 26%) accounted for most participants, followed by Asia (*n* = 145, 18%), Latin America and the Caribbean (*n* = 124, 15%), and North America (*n* = 110, 13%).

We found that respondents from Africa reported higher awareness of HIV interventions compared to participants from other regions (Fig. 1). Participants in Africa had higher awareness of HIV self-testing (64%, *n* = 132) than participants in Latin America and the Caribbean (59%, *n* = 68), Europe (51%, *n* = 107), North America (48%, *n* = 47), and Asia (37%, *n* = 52) (*p* < .001). PEP awareness was highest among respondents in Africa (75%, *n* = 151) and North America (75%, *n* = 74) compared to respondents in Latin America and the Caribbean (60%, *n* = 70), Europe (50%, *n* = 106), and Asia (42%, *n* = 58) (*p* < .001). Awareness of PrEP was highest in North America (75%, *n* = 73), followed by Africa (68%, *n* = 138) Latin America and the Caribbean (55%, *n* = 63), Europe (47%, *n* = 97), and Asia (40%, *n* = 55) (*p* < .001).

**FIG. 1.**
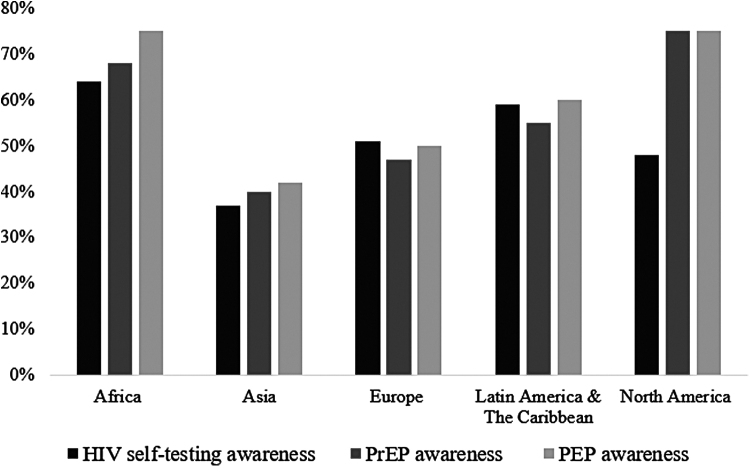
Geographical comparison of HIV intervention awareness. Regional differences in awareness of self-care interventions were statistically significant at *p* < .001 with Pearson's chi-squared test. PEP, post-exposure prophylaxis; PrEP, pre-exposure prophylaxis.

We observed that African respondents participating in the WHO's global values and preferences survey respondents had high awareness of HIV interventions relative to respondents in other regions. It is unclear from our study why this would be the case, but countries in Africa where these interventions have largely been deployed by various international donors benefit from a greater awareness of them. On the other hand, regions that do not have a high burden of HIV have no urgent need to be aware of HIV self-testing and PrEP and PEP, which could explain lower awareness outside of Africa. Regardless of why we observed a difference in awareness between Africa and other global regions, our findings suggest an opportunity to expand self-care interventions for HIV prevention and management in the Africa region, where the high awareness of self-care interventions can be leveraged to address the high HIV burden.

High awareness of self-care interventions bodes well for African health care systems amidst the COVID-19 pandemic, during which individuals might be more reluctant to access health facilities.^8^ Increased access to preventative and diagnostic self-care interventions for HIV could make it easier for individuals to know their status and simultaneously reduce their risk of acquiring HIV and COVID-19 (through avoiding potential contact with the coronavirus at health facilities). Health systems could improve health access and coverage by linking end users to self-care interventions through community-based practices; for example, by considering training standalone community pharmacists to provide HIV self-care interventions based on evidence that health care users find pharmacies more accessible, convenient, and time efficient than health facilities.^9^ For these reasons, community pharmacies have advocated for expanding PrEP and PEP access, and successfully pilot tested distributing HIV self-test kits.^10,11^

We acknowledge certain limitations that affected the internal and external validity of our findings. Primarily, our sample size was limited by the number of people who agreed to participate in the online survey. Second, since most respondents could access the survey through the WHO website or by being on a sexual and reproductive health listserv, it is likely that our sample would have had better knowledge on HIV self-care interventions leading to selection bias. Finally, since the survey was self-administered online, it excluded illiterate participants and people without access to the internet, thus compromising the generalizability of our sample—particularly in global regions of low literacy and relatively poor internet access such as Africa.

Despite these limitations, our study makes a valuable contribution to the literature, which—to our knowledge—has not presented a global, side-by-side geographical comparison of awareness of HIV interventions among potential end users. We urge future studies to build on our results by testing the possible predictors of disparate geographical awareness of HIV interventions we observed.

This study highlighted a potential path to improving universal health coverage and access to HIV testing and prevention in the Africa region, which is imperative to reaching Sustainable Development Goal 3, which seeks to ensure health and well-being for all persons. However, we caution that the high awareness of HIV self-care interventions noted among African participants in our study does not imply high access. HIV programs in Africa must therefore be mindful to bridge any gap between awareness of HIV interventions and access to them. A rational delegation of tasks to other health occupations such as community-based health workers and pharmacists, particularly in low-resource settings and where HIV burden is high, could improve health outcomes for individuals and communities.
